# Preparation of Humidity-Sensitive Poly(Ethylene Glycol) Inverse Opal Micropatterns Using Colloidal Lithography

**DOI:** 10.3390/ma10091035

**Published:** 2017-09-05

**Authors:** Bing Yu, Hailin Cong, Zhen Yang, Shujing Yang, Yuezhong Wang, Feng Zhai, Yifan Wang

**Affiliations:** 1 Institute of Biomedical Materials and Engineering, College of Chemistry and Chemical Engineering, Qingdao University, Qingdao 266071, China; yubingqdu@yahoo.com (B.Y.); yangzhenqdu@163.com (Z.Y.); cym72@tom.com (S.Y.); qq1060977923@163.com (Yu.W.); gg104205@163.com (F.Z.); wangyifan@qdu.edu.cn (Yi.W.); 2 Laboratory for New Fiber Materials and Modern Textile, Growing Base for State Key Laboratory, College of Materials Science and Engineering, Qingdao University, Qingdao 266071, China

**Keywords:** poly(ethylene glycol), inverse opals, colloidal crystals, sensors

## Abstract

Humidity-sensitive poly(ethylene glycol) (PEG) inverse opals with micropatterns of 2 μm wide anti-swell-broken grooves were prepared using polystyrene (PS) colloidal crystals as templates and colloidal lithography. Monodisperse PS colloids were deposited in an ordered manner onto glass slides using a double-substrate vertical deposition method to form colloidal crystal templates. Poly(ethylene glycol) diacrylate (PEGDA) with photoinitiator was infiltrated into the interspaces of the colloidal crystals and photo-crosslinked by UV irradiation through a photomask. After removal the PS templates and unexposed PEGDA by tetrahydrofuran (THF), PEG hydrogel micropatterns with three-dimensional ordered porous structures were obtained. The band gaps of the PS colloidal crystals and corresponding PEG hydrogel inverse opals were measured by UV-VIS reflection spectrometer, calculated by Bragg law and simulated by Band SOLVE. The obtained PEG hydrogel inverse opal micropatterns can be used as sensors for humidity sensing due to absorption and desorption of moisture in the band gap structures. The sensor had a very reliable performance after repeated humidity sensing, and could be mass produced facilely with very low cost. The photopatterned anti-swell-broken grooves play an important role in the reliability of the sensors.

## 1. Introduction

In past decade, three-dimensional ordered porous materials have attracted great attention for wide potential applications in different areas such as band gap materials, biomedical materials, energy storage, and conversion materials [1,2]. Many methods have been reported to fabricate ordered porous materials, in which the replication of colloidal crystal templates is one of the most commonly used approaches [3,4]. The monodisperse micro- or submicrometer colloids (organic or inorganic) [5,6], usually adopt face-centered cubic (fcc) packing with ~26% voids in volume [7,8], into which the guest molecules and nanoparticles are easily infiltrated through different strategies [9,10]. The templates subsequently can be removed by sintering for organic or acidic etching for inorganic templates to acquire the ordered porous network, in which the spherical voids are periodic and interconnected via small interstitial channels [11,12,13].

A lot of ordered porous materials, including inorganic, organic, metallic, polymeric, and ceramic, have been successfully made using this method [14,15,16]. The products, which are the exact inverse replicas of the templates with pores distributed in a three-dimensionally ordered way, can cause Bragg diffraction of lights [17,18]. Both organic and inorganic ordered porous materials are very interesting for theoretical research and practical applications such as making optical filters, switches, photonic crystals and chemical sensors [19,20,21]. For example, Ozin et al. synthesized macroporous maghemite by infiltrating colloidal crystal with polyferrocenylsilane [22]. Zhang et al. synthesized the hierarchically ordered porous carbon via in situ self-assembly of polymer and silica microspheres, which could be used as a catalyst support [23]. Cao et al. prepared ordered porous NaCl and KCl crystals, which could be used as template to prepare the submicrometer spheres of liquid crystals [24]. Wang et al. prepared multi-responsive hydrogel microparticles with inverse-opal structure [25].

Traditional humidity sensors often use graphene oxide, reduced graphene oxide or SnO_2_ as detection devices, which response according to changes in the electrical signals under different humidity. Hydrogels are widely used in different types of sensors in many fields because of their behavior of absorption, desorption, and swelling when exposed to water vapor or other chemical substances. When the water molecules are deep in the hydrogel sample, the chain of polymers will spontaneously arrange for the new conformation, which will lead to changes in polymer electricity, optics and other physical properties. For example, Wiltzius et al. created a polyacrylamide inverse opal hydrogel structure to from a colloidal crystal template. This material can respond to various humidity conditions by shifting its optical reflection peak noticeably within the visible wavelength range [26]. Silk-fibroin inverse opals with different spectral positions of bistructural color reflection are invented and show great humidity-responsive color sensing abilities [27]. 

In this paper, poly(ethylene glycol) (PEG) inverse opals with micropatterns of 2 μm wide anti-swell-broken grooves were obtained using polystyrene (PS) colloidal crystal as template and colloidal photolithography. The anti-swell-broken grooves are some grooves without samples in the micropatterns which can prevent dilatation of the patterns from each other. The bad gap properties of the PS opals and corresponding PEG hydrogel inverse opals were investigated using UV-VIS reflection spectrometer, as well as theoretical calculation and simulation. Under different humidity, the maximum band gaps of the PEG hydrogel inverse opal micropatterns shifted and showed different structural colors, and the band gap wavelength increased linearly with the increase of humidity, which could be used as sensors for humidity sensing. The performance of the sensors with and without micropatterns of anti-swell-broken grooves was studied and discussed preliminarily.

## 2. Experimental

### 2.1. Materials

Styrene (99%), methyl methacrylate (MMA, 99%), ammonium persulfate ((NH_4_)_2_S_2_O_8_, 98%), and ammonium bicarbonate (NH_4_HCO_3_, 99%) were purchased from Tianjin Chemical Company (Tianjin, China). 3-sulfopropyl methacrylate potassium salt (SPMAP, 98%) was purchased from Shanghai Jingchun Chemical Company (Shanghai, China). Poly(ethylene glycol) diacrylate (PEGDA) (M_n_ = 575 g/mol) and 2-hydroxy-2-methylpropiophenone were purchased from Sigma-Aldrich (Shanghai, China). Tetrahydrofuran (THF, 99%), hydrogen peroxide (30%), sulfuric acid (95~98%), and ethanol (99.5%) were purchased from Tianjin Chemical Company (Tianjin, China). Glass slides (75 mm × 25 mm × 1 mm) was purchased from Qingdao Chemical Company (Qingdao, China). Styrene and MMA were distilled under vacuum before use. The other reagents were used as received.

### 2.2. Synthesis of Monodisperse PS Colloids

Monodisperse PS colloids were synthesized with soap-free emulsion polymerization according to the literature with some modifications [28,29]. The typical procedure can be described as follows: 14.1 mL of styrene, 0.78 mL of MMA, 100 mL of deionized (DI) water were added into a three-necked flask under magnetic stirring. When the flask was heated to 70 °C, the first mixture consisting of 0.73 g of (NH_4_)_2_S_2_O_8_, 0.51 g of NH_4_HCO_3_, 0.12 g (or 0.05 g) of SPMAP, and 10 mL of DI water was added. Polymerization was carried out at 70 °C under nitrogen protection for 4 h, and then the second mixture consisting of 2.81 mL of styrene, 0.16 mL of MMA, 0.50 g of SPMAP, 0.10 g NH_4_HCO_3_, and 10 mL of DI water was added. Polymerization was continued for an additional 6 h at 70 °C to obtain white latex. After polymerization, the PS particles in the latex were separated by centrifugation at 12,000 rpm and washed with DI water for several times. Finally, the purified products were dried at 25 °C for 48 h. Monodisperse PS colloids with an average diameter of 190 nm (or 330 nm) were obtained.

### 2.3. Fabrication of PEG Inverse Opal Micropatterns

**Figure 1:**
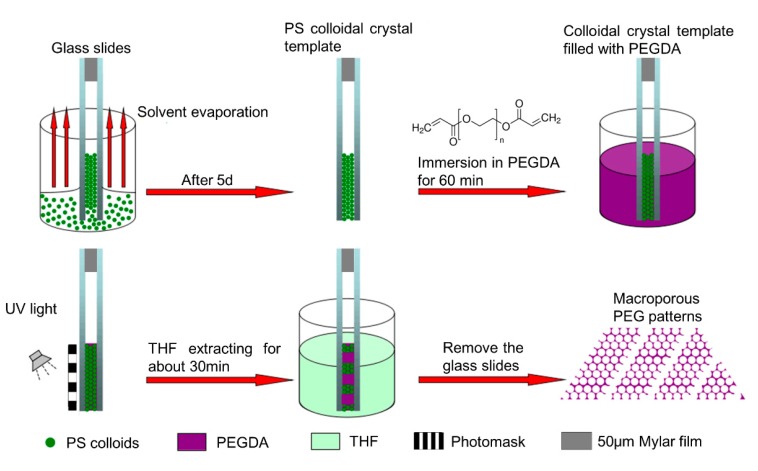
Schematic illustration of the fabrication process of PEG inverse opal micropatterns.

The PEG inverse opals was obtained as follows: glass slides (75 mm × 25 mm × 1 mm) were pretreated at 70 °C in H_2_SO_4_/H_2_O_2_ (7:3 V/V) for 30 min to create clean and hydrophilic surfaces. The substrates were finally rinsed with deionized water and dried with a stream of nitrogen. As show in Figure 1, PS colloidal crystal was obtained using the double-substrate vertical deposition method [27]. Two as-prepared glass slides were clamped together face to face with 50 μm Mylar films as spacers, and then were dipped vertically into a 50 mL beaker containing 10 mL of PS latex (3.4 mg/mL). Following the evaporation of the water, the PS colloids were packed orderly into a colloidal crystal under induction of the capillary force between the glass slides. After deposition for about five days, a colloidal crystal (~1.0 cm^2^) was formed between the glass slides. The PS colloidal crystal between glass slides was immersed into PEG prepolymer (PEGDA/2-hydroxy-2-methylpropiophenone, 30:1, V/V) for 60 min, and the infiltrated PEG prepolymer was photo-crosslinked using 2-hydroxy-2-methylpropiophenone as photoinitiator through a chrome/sodalime photomask to form PEG hydrogel under 365 nm UV light at an intensity of 10 mW/cm^2^ for 3 min. 2-Hydroxy-2-methylpropiophenone is a homolytic cleavage photoinitiator which can be excited from ground state when absorbs photons under ultraviolet light. The excited initiator then produce a Norrish Type I reaction, making the valence bond between adjacent C atoms in PEGDA elongated and fractured, generating primary reactive radicals, thereby leading to the photopolymerization. Compared with initiator-free photopolymerization [30], our approaches can improve the efficiency of the reaction and reduce the reaction time. The regions of PEG prepolymer exposed to UV light underwent free radical polymerization and became crosslinked. After immersing the formed PS/PEG hydrogel hybrid colloidal crystal between glass slides in THF for 30 min to remove the PS template and unexposed PEG prepolymer, ordered porous PEG hydrogel micropatterns (~1.0 cm^2^) were obtained.

### 2.4. Characterizations

A dynamic light scattering system (DLS, Dynapro Titan TC, Wyatt Technology, Goleta, CA, USA) was used to calculate polydispersity indices (PDI). The calculation is based on Equation (1), where Average represents the mean grain diameter of the particles. 

(1) $$\mathrm{PDI}=\sqrt{\frac{\mathrm{Standard}\;\mathrm{deviation}}{\mathrm{Average}}}$$

A scanning electron microscope (SEM, JSM-800, JEOL, Tokyo, Japan) was used to observe the diameter of PS colloids, the morphology of PS colloidal crystals, and the macroporous structure of PEG hydrogel micropatterns. The structural color of the PS colloidal crystal and PEG hydrogel inverse opal micropatterns were characterized using a UV-VIS reflection spectrometer (Ocean Optics, USB-2000, Dunedin, FL, USA) equipped with a 150 W haloid lamp cold light (Lamp-house, YN XD-301, South Yorkshire, UK). The UV-VIS spectra were collected under perpendicular irradiation of the cold light. The relative humidity (RH) was controlled and recorded by humidifier (Yadu, SC-X100J, Beijing, China) and hygrometer (KTJ, TA-218, Shenzhen, China), respectively. The structural color of PEG hydrogel inverse opal micropatterns was observed by a microscope (Cnmicro, SMZ-T1, Beijing, China) equipped with a digital camera. 

## 3. Results and Discussion

**Figure 2:**
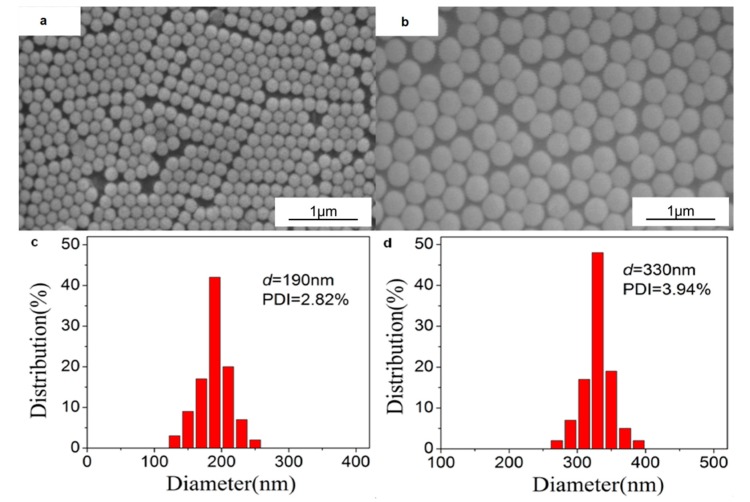
SEM images (**a**,**b**) and PDI analysis (**c**,**d**) of the PS colloids used to prepare colloidal crystal templates: (**a**,**c**) 190 nm; (**b**,**d**) 330 nm.

The size and distribution of the synthesized PS colloids visualized by SEM are shown in Figure 2. We can see that the two PS colloids with diameters of 190 nm and 330 nm are almost monodisperse with PDI less than 4%.

**Figure 3:**
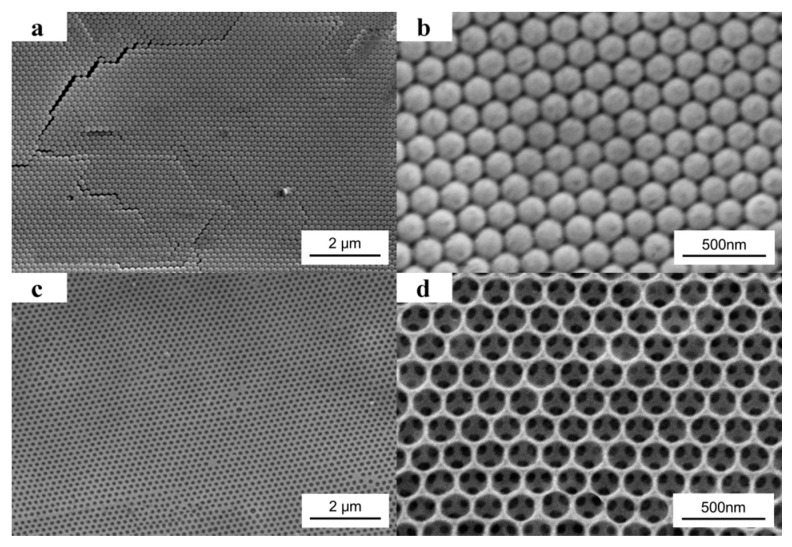
SEM images of 190 nm PS colloidal crystal template (**a**,**b**) and the obtained PEG hydrogel inverse opal without photomask (**c**,**d**) from the template.

SEM images of the 190 nm PS colloidal crystal template are shown in Figure 3a,b. Figure 3b is a higher magnification image of Figure 3a. From the planar image, we can see that the monodisperse PS colloids adopt a hexagonal array in the crystal, which is the (111) plane of the fcc packing. As shown in Figure 3c,d, after filled the template with PEG prepolymer, UV exposure without photomask, and removed the PS colloids by THF, the order porous PEG hydrogel inverse opal was obtained. Figure 3d is a higher magnification image of Figure 3c. From Figure 3d, a triangular pattern below every hole can be visualized. It is because that along the (111) normal direction, each hole is just located above three holes of the subjacent layer. From Figure 3a,c, few defects can be visualized, which indicates the double-substrate vertical deposition method based on capillary force is very successful in making the highly ordered structures.

The maximum band gaps of the colloidal crystal can be calculated according to the Bragg law shown in Equation (2) [31], where λ is the wavelength of the band gap in the (111) direction, k is an arbitrary integer coefficient, D is the diameter of colloids, n is the refractive index of the colloidal crystal, and θ is the angle of incidence.

(2) $$\begin{array}{l} k\lambda =2\sqrt{2/3}D\sqrt{n^{2}{-\sin}^{2}\theta }\\ k=1,2,3,\ldots \end{array}$$

In our experiment, incident light is vertical to the (111) surface, so the angle of incidence is zero (θ = 0). The refractive index (n) of the colloidal crystal and inverse opals can be calculated according to Equations (3) and (4) [32], respectively, where Φ represents the void ratio of the colloidal crystal (~26%), n_PS_, n_Air_ and n_PEG_ represent the refractive indices of the PS colloids (~1.59), air (~1.00), and PEG (~1.47), respectively.

(3) $$n^{2}={{(1-\Phi )n}_{\mathrm{PS}}}^{2}{{+\Phi n}_{\mathrm{Air}}}^{2}$$

(4) $$n^{2}={{(1-\Phi )n}_{\mathrm{Air}}}^{2}{{+\Phi n}_{\mathrm{PEG}}}^{2}$$

**Figure 4:**
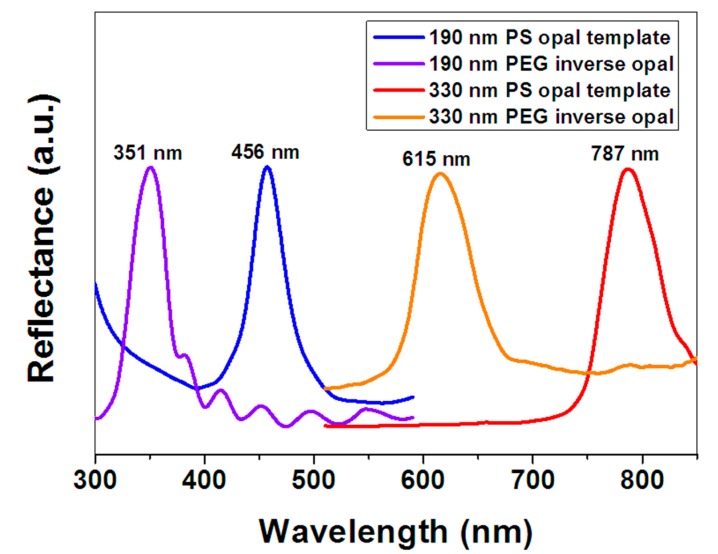
UV-VIS reflection spectra of PS opals and corresponding PEG hydrogel inverse opals.

Figure 4 shows the UV-VIS reflection spectra of the PS opals and corresponding PEG hydrogel inverse opals measured at normal incidence. We can see the band gaps of 190 nm and 330 nm PS opals are measured at 456 nm and 787 nm, respectively, which are very close to the calculated values (452.9 nm and 786.6 nm) according to Equations (2) and (3). The band gaps of 190 nm and 330 nm PEG hydrogel inverse opals are measured at 351 nm and 615 nm, respectively, which are very close to the calculated values (354.0 nm and 614.8 nm) according to Equations (2) and (4). This indicates that the structural colors coming from Bragg diffraction are sensitive to the diameters of PS colloids or PEG pores.

**Figure 5:**
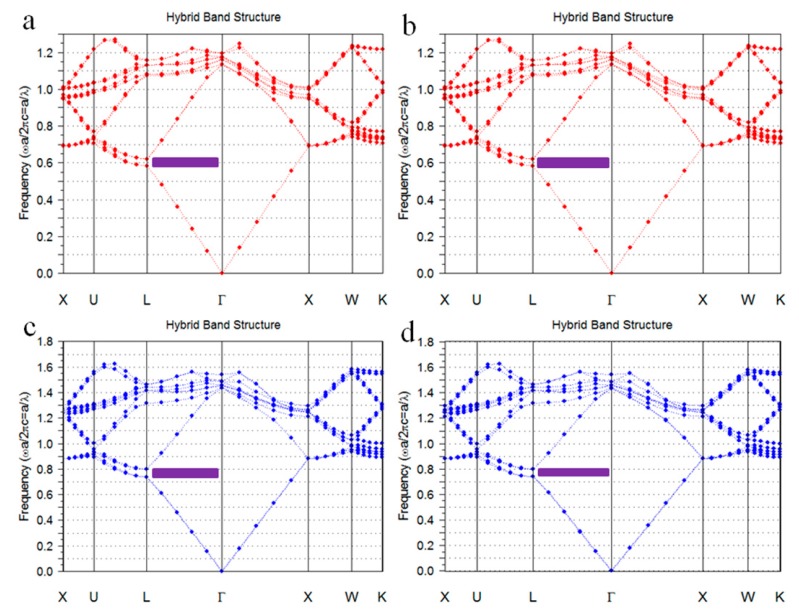
Band structures of PS colloidal crystals and PEG inverse opals: (**a**) 190 nm PS colloidal crystals; (**b**) 330 nm PS colloidal crystals; (**c**) 190 nm PEG inverse opals; and (**d**) 330 nm PEG inverse opals.

The band structures of colloidal crystals and inverse opals were simulated by commercial program (Band SOLVE, Rsoft design group, Tucson, AZ, USA). In the simulation, seven layers fcc structure was calculated, calculation steps were 16 and incidence angle was 0°, the refractive index of PS and PEG were 1.59 and 1.47, respectively. Figure 5a,b show the band structures of the PS colloidal crystals. The purple regions show the partial band gaps of fcc structure at Γ-L direction. The electromagnetic waves of the corresponding bang gap wavelength in these regions are prohibited from being propagated in the crystals along the 111 directions, which presented as reflected light. The reduced frequency (ω) of the partial band gaps ranges from 0.59 to 0.63. The bang gap wavelength λ = α/ω, where α is the periodic constant of crystal. For the fcc structure crystal, the relationship between α and microsphere diameter D is α = 1.414 D. As can be seen from Figure 5a,b, the reduced frequency (ω) of the band gap of PS colloidal crystals was 0.59 to 0.63. Accordingly, the band gaps of the colloidal crystals formed by 190 nm and 330 nm PS microspheres with fcc structures range from 426 to 455 nm and 741 to 791 nm, respectively. Figure 5c,d show the band structures of the inverse opals. The reduced frequency of the partial band gaps ranges from 0.75 to 0.81. Accordingly, the band gaps of the PEG inverse opals formed from 190 to 330 nm PS opals with fcc structures range from 332 to 358 nm and 576 to 622 nm, respectively. Since the location of the reflection peak of each sample in Figure 4 exactly matches the band gap range calculated in Figure 5, the simulated band structures of colloidal crystals and inverse opals shown in Figure 5. Agree well with the experimental results shown in Figure 4.

**Figure 6:**
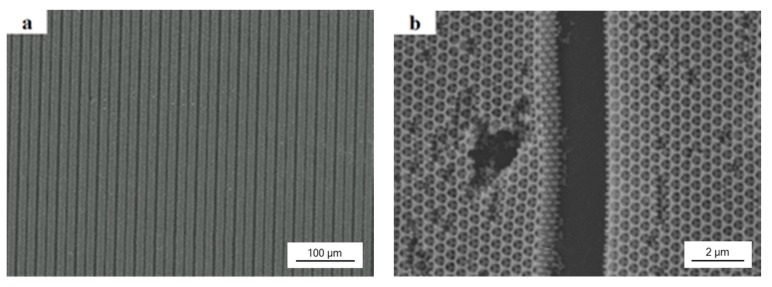
SEM images of 330 nm PEG hydrogel inverse opal micropatterns with 2 μm wide anti-swell-broken grooves: (**a**) low-magnification image; (**b**) high-magnification image.

The obtained PEG hydrogel inverse opals could be used as sensors for humidity sensing. In order to avoid broken by swell in humidity environment, we did the colloidal lithography with a photomask to make 2 μm wide anti-swell-broken grooves among 10 μm wide PEG hydrogel inverse opals. As shown in Figure 6a,b, after filled the colloidal crystal template with PEG prepolymer, UV exposure through a photomask, and removed the PS colloids and unexposed PEG prepolymer by THF, the order porous PEG hydrogel inverse opal micropatterns with a minimum resolution of 2 μm were obtained. Figure 6b is a higher magnification image of Figure 6a.

**Figure 7:**
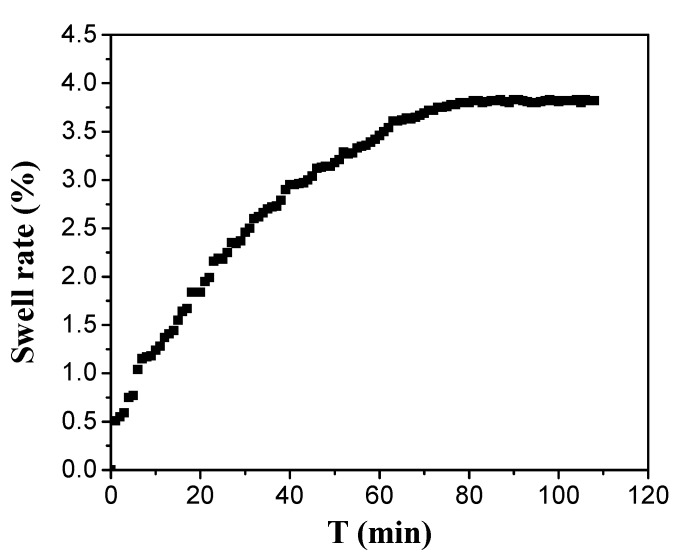
Swell curve of 330 nm PEG hydrogel inverse opal micropatterns in 30% humidity.

As shown from Figure 7, the structure of PEG hydrogel inverse opal micropatterns can expand in 1 min and reach stability after 70 min under low humidity environment. Thus, we demonstrated our PEG hydrogel inverse opal micropatterns has a fast response to humidity change and takes a long time to stabilize the humidity response. 

**Figure 8:**
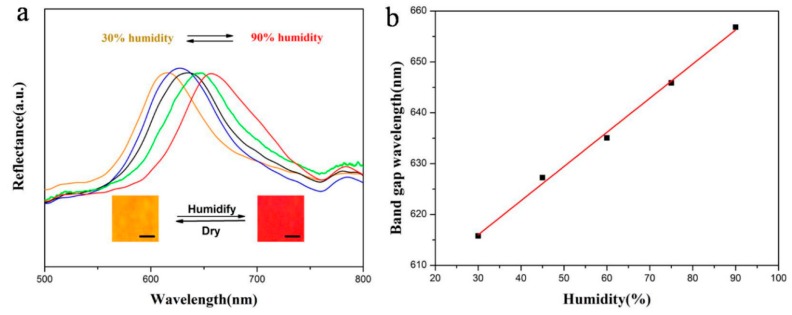
Application of 330 nm PEG hydrogel inverse opal micropatterns as a humidity sensor: (**a**) UV-Vis reflection spectra at different humidity (Insets show the photos of the inverse opal sensors took at 30% and 90% humidity, scale bars: 200 μm); and (**b**) relationship of the band gap wavelength and humidity.

As shown in Figure 8, when the relative humidity increases from 30% to 90%, the structural color of the 330 nm PEG hydrogel inverse opal micropatterns change from orange to red, and red shift of the band gaps can be seen obviously in the whole process. The band gap wavelength increases linearly with the increase of humidity. According to Equation (1), the wavelength of the band gap is related to the diameter D of the reflector. For PEG hydrogel inverse opal micropatterns after removing the template, when the humidity increases, the PEG will swell in a certain degree, leading to the increase of D. As a result, the band gaps are red shifted. Additionally, the color variation is reversible due to the absorption and desorption of moisture in the ordered porous hydrogel structures under the humidifying and drying conditions. Compared with the traditional electrical humidity sensors, the PEG hydrogel detector is not only economic, but also a reversible naked-eye sensor.

**Figure 9:**
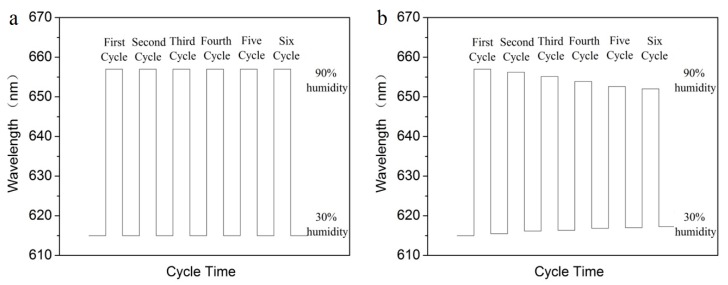
Band gap property of the PEG hydrogel inverse opal sensors with (**a**) and without micropatterns of anti-swell-broken grooves (**b**) after six cycles of humidity sensing.

As shown in Figure 9a, after six cycles of humidity sensing from 30% to 90%, there is no obvious change in the band gap property of the sensors with micropatterns of anti-swell-broken grooves, which indicates the PEG hydrogel inverse opal micropatterns has a very reliable performance, and can be used repeatedly for humidity sensing. Compared with the PEG hydrogel inverse opal without micropatterns of anti-swell-broken grooves (Figure 9b), we can see that the photopatterned anti-swell-broken grooves play an important role in the reliability of the sensors. In high humidity, the structure of PEG inverse opal structure will expand. PEG hydrogel inverse opal sensors without anti-swell-broken grooves will be irreversibly damaged caused by excessive squeeze. While, PEG sensors with anti-swell-broken grooves can stay stable performance because of the anti-swell-broken grooves can play as a buffer to protect the inverse opal structure of PEG.

## 4. Conclusions

Monodisperse PS colloids of 190 nm and 330 nm were synthesized successfully using soap-free emulsion polymerization method. PS colloidal crystal templates were prepared with few defects from the PS colloids using a double-substrate vertical deposition method. After photo-crosslinking the infiltrated PEG prepolymer in the template through a photomask, and removal the PS colloids and unexposed PEG prepolymer by THF, PEG inverse opals with micropatterns of 2 μm wide anti-swell-broken grooves were fabricated successfully. The measured band gaps of the PS opals and PEG hydrogel inverse opals were in accordance with the theoretical calculation and simulation very well. The obtained PEG hydrogel inverse opal micropatterns could be used as sensors for humidity sensing. The band gap wavelength increases linearly with the increase of humidity. The color variation is reversible due to the absorption and desorption of moisture in the ordered porous hydrogel structures under the humidifying and drying conditions. The sensor had a very reliable performance after repeated humidity sensing, and could be mass produced facilely with very low cost. The photopatterned anti-swell-broken grooves play an important role in the reliability of the sensors.
